# Supplementary material for: Efficient, biosafe and tissue adhesive hemostatic cotton gauze with controlled balance of hydrophilicity and hydrophobicity
Source: Nat Commun. 2022 Jan 27;13:552. doi: 10.1038/s41467-022-28209-8 (PMC8795195; doi:10.1038/s41467-022-28209-8)
Supplement: Supplementary file 1 — Supplementary Information [file 41467_2022_28209_MOESM1_ESM.pdf]

Supplementary Information

# A super-efficient and biosafe hemostatic cotton gauze with controlled balance of hydrophilicity/hydrophobicity and tissue adhesiveness

Huaying He[1], Weikang Zhou[1,2], Jing Gao[1], Fan Wang[3], Shaobing Wang[3], Yan Fang[1*], Yang Gao[1], Wei Chen[1,4*], Wen Zhang[1], Yunxiang Weng[1], Zhengchao Wang[3] & Haiqing Liu[1,4*]

## Content

1. Synthesis of ABO and USO-g-gauze ... 3
   (1) Synthesis of 4-allyl-1,2-benzenediol (ABO) ... 3
   (2) Optimization of synthetic conditions for USO-g-gauze ... 4
2. FTIR spectra of gauze samples ... 5
3. XPS analysis of gauze samples ... 5
4. SEM of gauze samples ... 7
6. Hemostatic performance of USO grafted chitosan gauze (USO-g-chitosan) ... 8
   (1) Preparation of USO-g-chitosan ... 8
   (2) Hemostatic performance on rat femoral artery injury ... 8
7. Hemostatic performance of Surgicel® gauze on rat liver laceration model ... 10
8. Rat survival rate in the injury models ... 10
9. Hemostasis in the pig skin laceration model ... 11
10. Theoretical insight into the tissue adhesiveness of USO-g-gauze ... 11
11. Peeling force of gauzes on fresh wet rat femoral tissue ... 18
12. Inactivation of catechol group on USO-g-gauze and their hemostatic performance 18
13. Morphology of Bandage® ... 21
14. Growth and proliferation of L929 on gauze samples ... 21
Supplementary References ... 24

1. Synthesis of ABO and USO-g-gauze

**(1)** Synthesis of 4-allyl-1,2-benzenediol (ABO)

ABO containing a catechol group was prepared according to the procedure shown in Supplementary Fig. 1.[1] Briefly, eugenol (8.6 mL, 56 mmol) and TES (19 mL, 112 mmol) were added to a 100 mL round bottom flask with a condenser and stirred at 24 °C for 5 min, then TPFPB (57 mg, 0.112 mol) was added into the mixture and reacted for another 3 h. Subsequently, the mixture was passed through a neutral alumina column with dichloromethane as the eluent to eliminate TPFPB. Then the dichloromethane and unreacted TES in the collected mixture were removed by a vacuum rotary evaporator at 60 °C. As-obtained eugenol protected by TES was present in a yellowish liquid. Finally, 5 mL of 0.1 M hydrochloric acid aq. solution was added and stirred at 80 °C for 6 h to de-protection for obtaining ABO. The chemical structure of TES protected eugenol and ABO was confirmed by $^{1}$H NMR analysis.

$^{1}$H-NMR of TES protected eugenol：6.78 ppm (d, 1H, Ar-*H*), 6.69 ppm (s, 1H, Ar-*H*), 6.63 ppm (d, 1H, Ar-*H*), 5.98 ppm (m, 1H, -C*H*=$CH_2$), 5.06 ppm (m, 2H, -CH=C$H_2$), 3.34 ppm (d, 2H, -C$H_2$-CH=$CH_2$), 0.98 ppm (t, 18H, -Si-$CH_2$-C$H_3$), 0.73 ppm (q, 12H, -Si-C$H_2$-$CH_3$).

$^{1}$H-NMR of ABO：6.78 ppm (d, 1H, Ar-*H*), 6.71 ppm (d, 1H, Ar-*H*), 6.62 ppm (dd, 1H, Ar-*H*), 5.93 ppm (m, 1H, -C*H*=$CH_2$), 5.29 ppm (b.s., 1H, -O*H*); 5.21 ppm (b.s., 1H, -O*H*), 5.05 ppm (m, 2H, -CH=C$H_2$), 3.27 ppm (d, 2H, -C$H_2$-CH=$CH_2$).

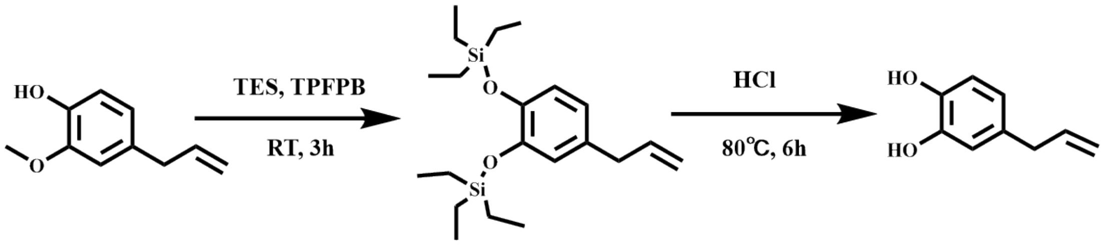


**Supplementary Figure 1.** Preparation scheme of 4-allyl-1, 2-benzenediol (ABO).

**(2)** Optimization of synthetic conditions for USO-g-gauze

Preliminary experiments to optimize the synthesis conditions were conducted. As listed in Supplementary Table 1, four USO/ethanol solutions with USO concentration of 0.5, 1.0, 2.0 and 2.5% were used to make USO-g-gauze. The graft amount ranged from 0.02 wt% to 0.28 wt% increasing with USO concentration. Water droplet instantly permeates and spreads in #1 and #2 gauzes, while it steadily stands on #4 within 60 s with WCA of ca. 120 (Supplementary Fig. 2). #3 gauze has a unique wetting property, as detailed in the main text. In a pre-evaluation of their hemostatic ability in the rat femoral artery injury model, the hemostatic performance of #1 and #2 gauze is similar to that of standard cotton gauze, while #4 gauze is similar to HTMS-g-gauze. Therefore, #3 USO-g-gauze was prepared and used for the systematic study shown in this research work.

**Supplementary Table 1**. WCA of a series of USO-g-gauze

| USO-g-gauze | USO conc. in USO/ethanol solution (wt%) | Graft amount by weight (wt%) | WCA (°) at time of 0 s | WCA (°) at time of 60 s |
|---|---|---|---|---|
| #1 | 0.5 | 0.02 | 0 | 0 |
| #2 | 1.0 | 0.04 | 0 | 0 |
| #3 | 2.0 | 0.10 | 68.2 ± 2.1 | 0 |
| #4 | 2.5 | 0.28 | 120.5 ± 3.6 | 120.2 ± 3.4 |

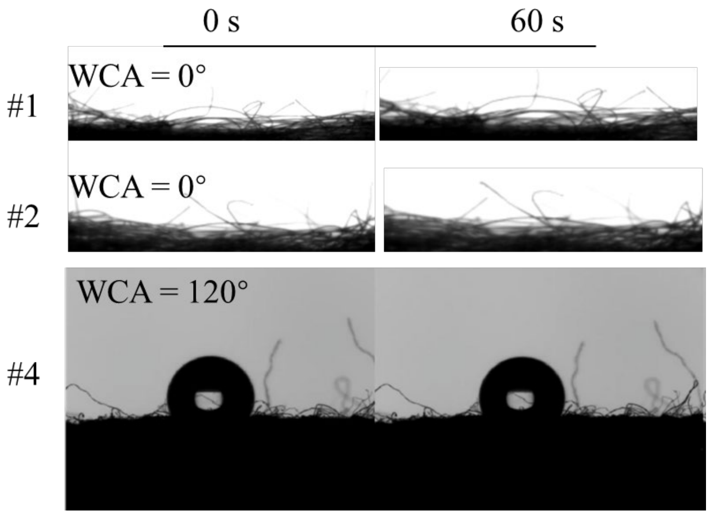


**Supplementary Figure 2.** WCA of #1, #2, #4 USO-g-gauze at 0 s and 60 s.

2. FTIR spectra of gauze samples

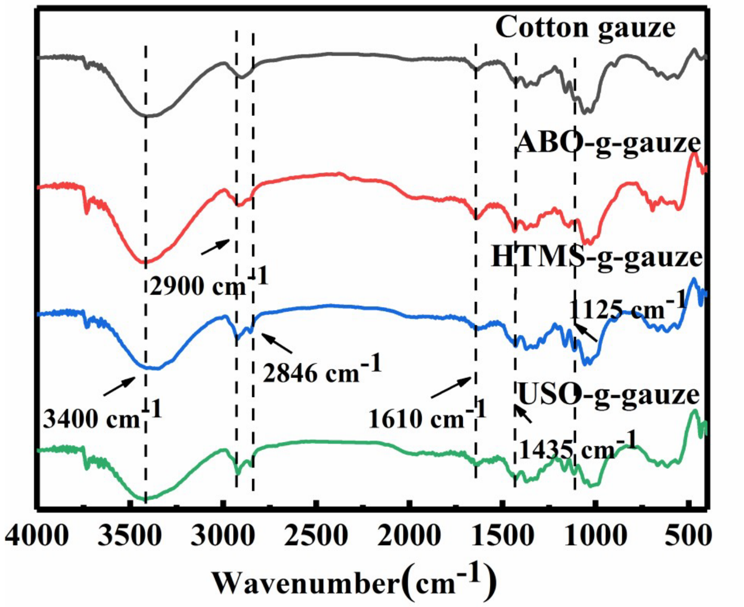


**Supplementary Figure 3.** FTIR spectra of gauze samples.

3. XPS analysis of gauze samples

XPS spectra of cotton gauze and USO-g-gauze are shown in Supplementary Fig. 4. In the survey scan spectrum of the gauze, there are two distinct peaks of $C_{1s}$ (286.40 eV) and $O_{1s}$ (532.40 eV). An additional peak for $N_{1s}$ (400.08 eV) is shown for USO-g-

gauze (Supplementary Fig. 4a). It is introduced by the low temperature plasma treatment in $N_2$ atmosphere.[2]

Based on the $C_{1s}$ high-resolution scan spectra (Supplementary Fig. 4b), the C/O ratio of cotton gauze and USO-g-gauze is 1.9:1 and 2.5:1, respectively. The increasing C/O ratio of USO-g-gauze is due to that the grafted USO contains an alkyl chain with 15 carbons. The $C_{1s}$ signal is fitted into three categories, i.e. the three peaks at 284.96, 286.75, and 288.0 eV are respectively attributed to the carbon of C-C, C-O-H, and O-C-O/C=O bond of cellulose. For USO-g-gauze, the $C_{1s}$ peaks for C-C, C-O-H, and O-C-O/C=O bond appear at very similar locations: 284.68, 286.46, and 288.23 eV, respectively. However, the relatively strongest peak of USO-g-gauze is C-C bond, rather than that of cotton is C-O-/C-OH bond. This is in accordance with the abundance of C-C bond from the long aliphatic chain grafted on USO-g-gauze.

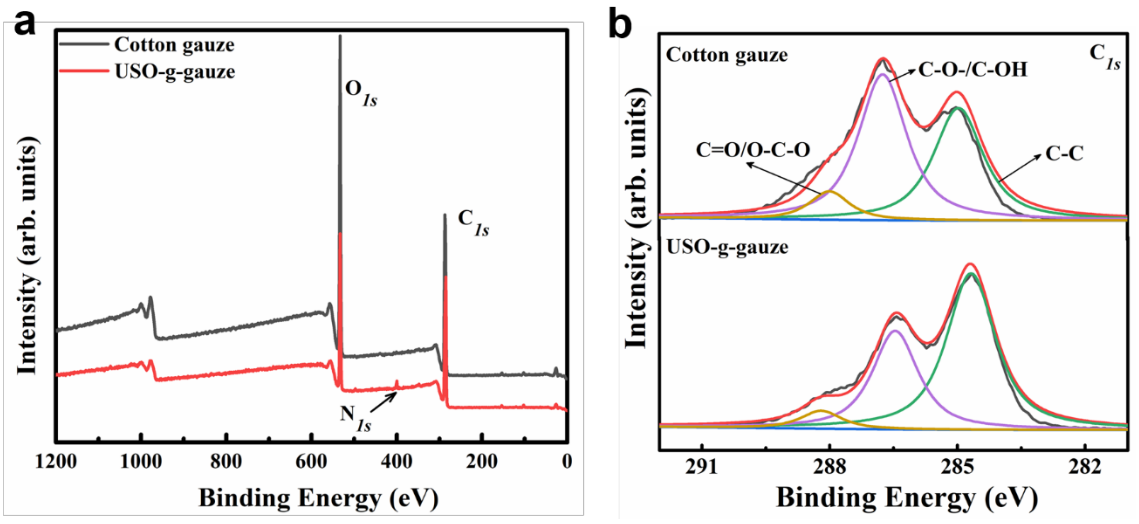


**Supplementary Figure 4.** XPS spectra. (a) XPS survey and (b) $C_{1s}$ high resolution scan spectra of cotton gauze and USO-g-gauze.

4. SEM of gauze samples

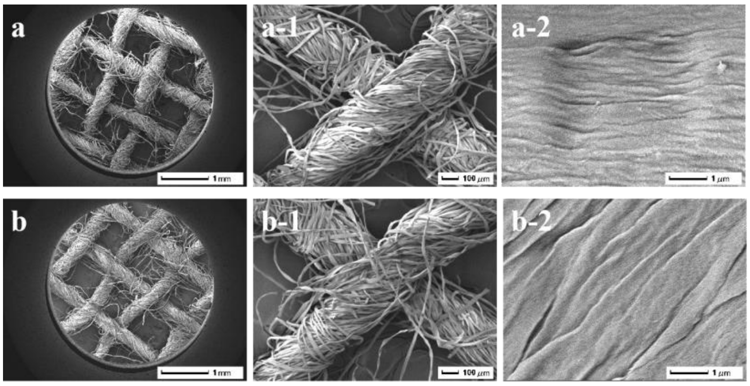


**Supplementary Figure 5.** SEM images. (a) ABO-g-gauze and (b) HTMS-g-gauze at different magnifications. Five spots were observed independently with similar results.

5. Moisture management test of gauzes

The movement of water in gauze was quantitatively measured by moisture management test (MMT). The wetting time (top side) is as short as 0.19 s for cotton and ABO-g-cotton gauzes, and substantially increases to 1.59 s for USO-g-cotton, and to 15.52 s for hydrophobic HTMS-g-gauze. As indicated in Supplementary Table 2, the shorter wetting time is, the faster the water absorption rate and spreading speed is. The water absorption rate of gauzes follows a decreasing order of Cotton gauze > ABO-g-gauze > USO-g-gauze > HTMS-g-gauze, while the water spreading speed is in the order of Cotton gauze ≈ ABO-g-gauze >> USO-g-gauze >> HTMS-g-gauze. The changing patterns of these indices suggest that the surface chemical structure of gauze effectively guides moisture movement (wetting, spreading, and diffusion) in gauze. The designed concomitant hydrophobic/hydrophilic structure of USO-g-cotton imparts it with not only proper wetting time and spreading rate, but its ability of water diffusion from one side to the other side (indicated by the cumulative one-way transport capacity, Supplementary Table 2). Such a unique property would be very

helpful for controlling blood movement in gauze and at the gauze/tissue contact surface when it is practically applied as a topical hemostat, as will be shown in the following sections.

**Supplementary Table 2.** Results of moisture management test of gauzes

| Sample | Wetting time (s) | | Maximum wetted radius (mm) | | Water absorption rate (%/s) | Spreading rate (mm/s) | Cumulative one-way transport capacity |
|---|---|---|---|---|---|---|---|
| | Top side | Bottom side | Top side | Bottom side | Top side | Top side | |
| Cotton gauze | 0.19±0.00 | 0.19±0.00 | 20.00±5.00 | 20.00±5.00 | 28.52±2.85 | 20.19±1.41 | 220.75±54.12 |
| ABO-g-gauze | 0.19±0.00 | 0.19±0.00 | 10.00±0.00 | 10.00±0.00 | 11.43±5.20 | 18.03±0.10 | 370.45±21.40 |
| HTMS-g-gauze | 15.52±0.58 | 48.66±5.64 | 5.00±0.00 | 3.33±0.35 | 3.07±0.16 | 0.32±0.01 | 843.65±56.36 |
| USO-g-gauze | 1.59±0.57 | 3.00±0.00 | 10.00±0.00 | 5.00±0.00 | 9.33±3.50 | 3.41±0.64 | 672.02±68.44 |

Data are shown as mean ± standard deviation (SD), $n = 3$

6. Hemostatic performance of USO grafted chitosan gauze (USO-g-chitosan)

**(1)** Preparation of USO-g-chitosan

A swatch of chitosan nonwoven was soaked in aq. acetic acid solution (pH 5.5) for 15 min, then put in 0.1 wt% sodium alginate (SA) aq. solution for 30 min at room temperature. Subsequently, the swatch was taken out and washed away free SA with distilled water, and completely dried at 60 °C. Then USO was grafted onto it through the same procedure for USO-g-gauze.

**(2)** Hemostatic performance on rat femoral artery injury

Chitosan nonwoven (Supplementary Fig. 6a) (thickness: ca. 2 mm) is composed of microfibers with diameter of ca. 20 μm (Supplementary Fig. 6b). Its chemical structure features -$NH_2$ groups in the anhydroglucose repeating unit, and it is a good source for efficient hemostatic products.[3, 4] USO is grafted onto chitosan fibers (Supplementary Fig. 6c). Two gauze swatches stacked layer by layer (Supplementary Fig. 6d) are compressed onto the transected rat femoral artery (Supplementary Figs. 6e, f) for 5 s, followed by static covering for a total time of 180 s (Supplementary Figs. 6g, h). The

gauzes are then removed to check the injuries (Supplementary Figs. 6i, j). It is seen that re-bleeding occurs for the injury treated with chitosan gauze (Supplementary Fig. 6k, pointed by the yellow arrow), all four sides of the two swatches are blood-wetted because of blood absorption and diffusion (Supplementary Fig. 6m), while the injury treated with USO-g-chitosan is relatively clean (Supplementary Figs. 6J, L) and only a small area on the first side is wetted with blood, even without diffusion to the second side (Supplementary Fig. 6n). The blood loss for USO-g-chitosan is just 5.7% of that for chitosan gauze (Supplementary Fig. 6o). The hemostatic efficiency of USO-g-chitosan is well demonstrated in Supplementary Movie 4.

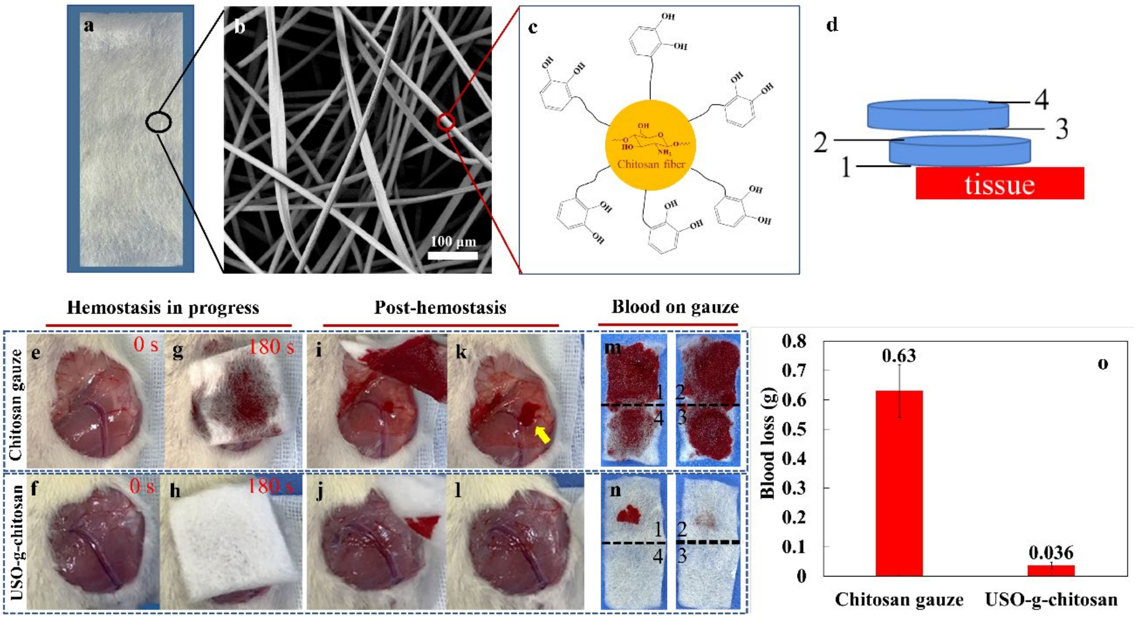


**Supplementary Figure 6.** USO-g-chitosan nonwoven gauze. (a) a digital photo and (b) a SEM image of USO-g-chitosan nonwoven gauze, (c) a cartoon for the chemical structure of USO-g-chitosan, (d) a cartoon for applying two gauze swatches stacked layer by layer on the bleeding tissue, the numbers indicate the side of the gauze, (e, f) rat femoral artery, (g) chitosan gauze and (h) USO-g-chitosan on a bleeding artery for 180 s, (i-l) gauzes were removed after hemostasis to check the wounds, (m, n) blood absorption and diffusion in gauzes, the numerical number indicates the gauze side as shown in D, (o) blood loss for chitosan and USO-g-chitosan gauze. Data in O are shown as mean ± SD, $n$ = 4, error bars represent SD. Source data for O are provided as a Source Data file. Image of b: ten spots were observed independently with similar results.

7. Hemostatic performance of Surgicel® gauze on rat liver laceration model

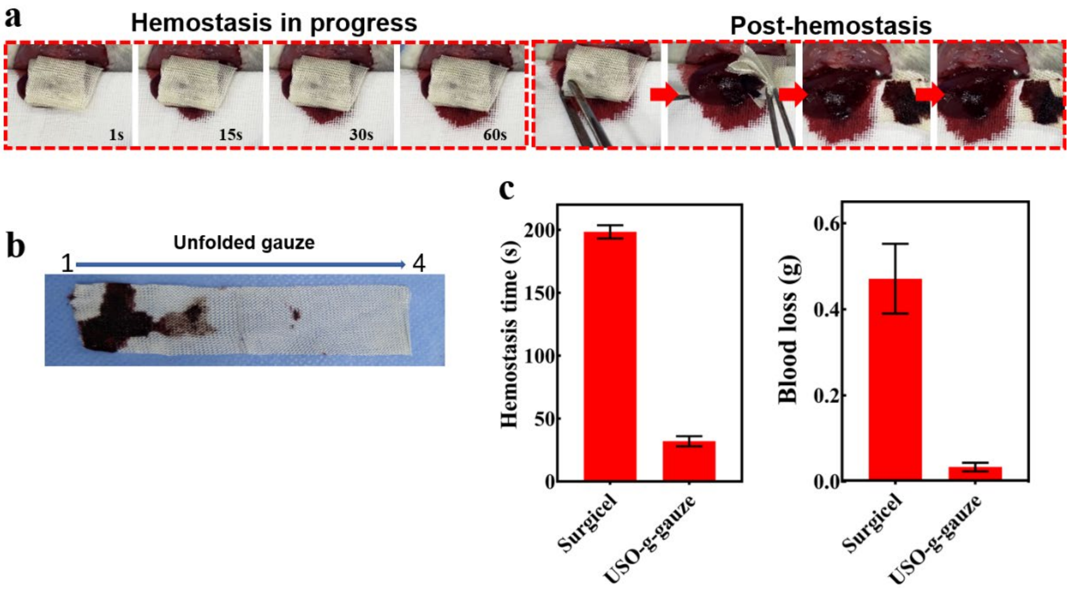


**Supplementary Figure 7.** Hemostatic performance of Surgicel on rat liver injury. (a) Hemostatic photos, (b) unfolded gauze, (c) hemostatic time and blood loss. Data in c are shown as mean ± SD, $n = 6$, error bars represent SD. Source data for c are provided as a Source Data file.

8. Rat survival rate in the injury models

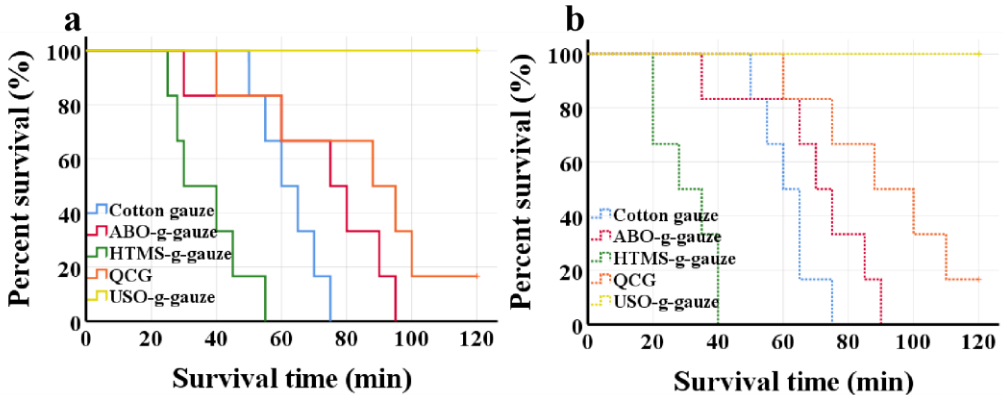


**Supplementary Figure 8.** Survival rates of the rats treated with gauzes. (a) femoral artery injury and (b) liver injury models treated with gauzes.

9. Hemostasis in the pig skin laceration model

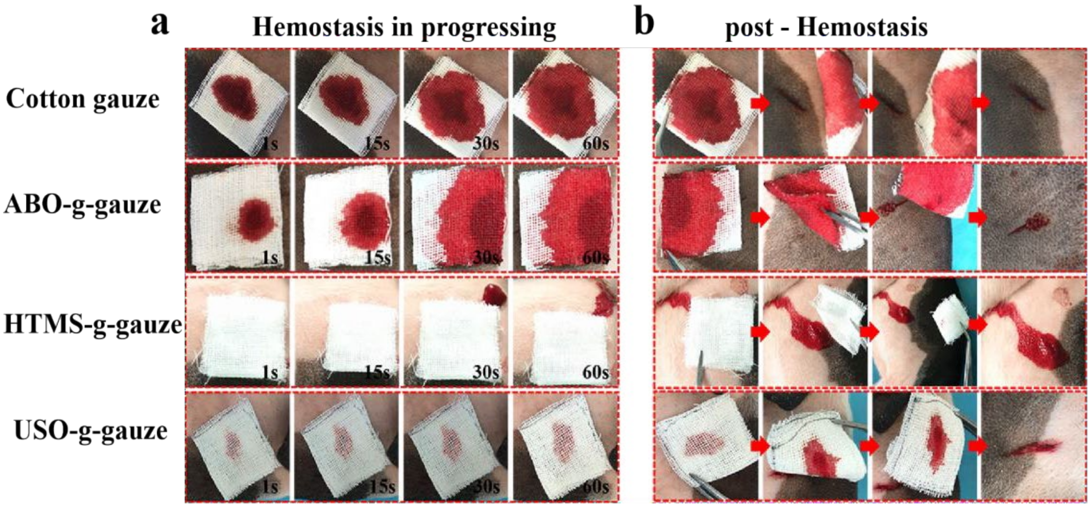


**Supplementary Figure 9.** Hemostasis in the pig skin laceration model. (a) Progressing of hemostasis after gauzes were put on wound, and (b) Removing gauze from wound after hemostatic state reached.

10. Theoretical insight into the tissue adhesiveness of USO-g-gauze

The generalized gradient approximation (GGA) with the Perdew–Burke–Ernzerh (PBE)[5] of the exchange correlation functional (including a semi-empirical van der Waals (vdW)[6] correction to account for the dispersion interactions) and a 400 eV cutoff for the plane-wave basis set are adopted to perform all the density functional theory (DFT)[7] computations of the studied systems within the frame of the Vienna ab initio simulation package (VASP)[8, 9]. The projector-augmented plane wave (PAW) is used to describe the electron-ion interactions[10, 11]. The atomic relaxation is performed until the total energy variation is smaller than $10^{-4}$ eV and all forces on each atom are 0.02 eV $Å^{-1}$.

Moreover, the following equation is defined to calculate the adsorption energies of the amino acid molecules on the modified cellulose with the alkyl chain terminated with catechol:

$$\Delta E_{ads} = E_A + E_B - E_{AB} \qquad (1)$$

Where $E_A$, $E_B$ and $E_{AB}$ are the total energies of the alkyl-chain-modified cellulose, the

sole amino acid molecule, and the composite system, respectively.

Initially, the geometric structure of cellulose has been fully optimized, as show in Supplementary Fig. 10a. Its optimized lattice parameters are $a$ = 7.72, $b$ = 8.45 and $c$ = 21.97Å, respectively, all of which are close to the corresponding experimental results[12]. Subsequently, we construct the structural model of composite system, namely the modified cellulose structure with the alkyl chain containing fifteen carbon atoms in the skeleton and having the terminal linked with catechol. Specifically, based on the optimized cellulose structure, we employ the structural unit as the theoretical model to simulate the structure of cellulose, and then connect the optimized alkyl chain to the surface of cellulose by substituting the H atom of the hydroxyl group, as illustrated in Supplementary Fig. 10b. We have fully optimized the structure of this model, and named it CAC for convenience. Meanwhile, we also optimized 16 different kinds of amino acid molecules (Supplementary Figs. 10c, d), which can be used as important components of the peptide chain in the protein, including alanine (A), aspartic acid (D), glutamic acid (E), phenylalanine (F), glycine (G), isoleucine (I), lysine (K), leucine (L), methionine (M), asparagine(N), glutamine (Q), arginine (R), serine (S), threonine (T), valine (V) and tyrosine (Y).

Ultimately, based on the structural model of CAC, we carried out the DFT calculations to investigate the adsorption properties of the alkyl-chain-modified cellulose system for the correlative amino acid molecules, by considering the possible adsorption configurations. We calculated the adsorption energies ($\Delta E_{\mathrm{ads}}$) of amino acid molecules on the CAC system by the formula (1), where the possible interaction modes were considered, such as the π-π stacking interaction and hydrogen bond interaction.

After relaxation, we can find that the amino acid molecules Y and F with the benzene ring can ultimately be adsorbed on the catechol at the end of alkyl chain via the π-π

stacking interaction with the distance between two rings as 3.424 Å and 3.408 Å respectively, as shown in Supplementary Fig. 10c and Supplementary Table 3. At the same time, the hydrogen bonding interaction can be also observed between them, where the H atom in a OH group of catechol can effectively interact with the lone pair of N atom in $NH_2$ group in amino acid molecule Y or F, with the interaction distance about 1.720 Å, as illustrated in Supplementary Fig. 10c. All of these can be also supported by the distribution of the correlative electron densities and the calculated electrostatic potentials (Supplementary Fig. 10c). As a result, the computed $\Delta E_{ads}$ values for F and Y are as large as 0.621 and 0.729 eV (Supplementary Fig. 10c), respectively, indicating that these two amino acid molecules containing the π-conjugated-ring can be effectively adsorbed on the catechol of CAC structure through the synergistic action of π-π stacking and hydrogen bonding.

Comparatively, all the remaining fourteen amino acid molecules, including A, D, E, G, I, K, L, M, N, Q, R, S, T and V, can effectively interact with the catechol of CAC structure by adopting two hydrogen bonds. It can be found that one hydrogen bond is formed by the H atom in COOH group of correlative amino acid molecules interacting with O in one OH group of catechol, while another hydrogen bond is formed through the O atom in COOH group interacting with H in another OH group of catechol, as presented in Supplementary Fig. 10d. This can be also supported by the calculated electrostatic potentials (Supplementary Fig. 10d). All these hydrogen bonding distances are in the range of 1.69 ~ 1.80 Å (Supplementary Table 3), and the computed $\Delta E_{ads}$ values are as large as 0.598, 0.622, 0.639, 0.617, 0.637, 0.637, 0.570, 0.636, 0.619, 0.637, 0.602, 0.592, 0.603 and 0.607eV for A, D, E, G, I, K, L, M, N, Q, R, S, T and V, respectively (Supplementary Fig. 10d), indicating the strong interaction between them.

Furthermore, we also explore the effect of relative position between two OH groups on the benzene ring on the adsorption energy of the sampled amino acid molecule G on the modified cellulose system, by considering three different configurations I~III, in which the two OH groups are separated by one, two or three H atoms, respectively, as shown in Supplementary Fig. 11. Our computed results reveal that when changing the relative position between the two OH groups from the original ortho- to meta- to para- arrangements, the $\Delta E_{ads}$ values for G can be reduced from 0.617 to 0.502/0.419 and then to 0.397/0.384 eV. Further, when the two OH groups are separated by three H atoms, the small adsorption energy (*ca.* 0.383/0.355eV) can be maintained. Clearly, with the increase of the spacing distance between the two OH groups, the adsorption energy for the sampled amino acid molecule decreases significantly, in view of the fact that two hydrogen bonds cannot be effectively formed at the same time or only single hydrogen bond can be formed (Supplementary Fig. 11). Therefore, the relative position between two OH groups on the benzene ring can have an important influence on the adsorption energy, where the ortho position can bring the maximum adsorption energy due to the formation of two hydrogen bonds.

Further, we also calculated the adsorption energy $\Delta E_{ads}$ of the sampled amino acid molecule G on the modified cellulose system with the alkyl chain terminated by phenol with one OH group, which can be considered as the corresponding structure by removing one of two OH groups in catechol of CAC. As shown in Supplementary Fig. 12, two configurations I and II can be obtained, where single hydrogen bond is observed between G and phenol with a distance about 1.804 and 1.753 Å, respectively. The computed $\Delta E_{ads}$ values are 0.248 and 0.355 eV, respectively, both of which can be about half of that of the corresponding structure with the two hydrogen bonds. This means that both the hydrogen bonds can be effectively formed simultaneously between

the relevant amino acids and catechol in CAC. Clearly, all of these can reflect the superior structural match between the amino acid molecules and the catechol used.

Overall, the modified cellulose structure with the alkyl chain can effectively interact with all these amino acid molecules via two hydrogen bonds or the synergistic action of π-π stacking and hydrogen bonding, indicating that this new type of hemostatic cotton fabric can exhibit excellent issue adhesiveness, where the catechol at the end of alkyl chain can play a crucial role.

**Supplementary Table 3.** The computed bond lengths ($d_{H\text{-}O}$) of the related hydrogen bonds as well as the distance ($d_{\pi\text{-}\pi}$) between two parallel benzene rings for all sixteen optimized structures CAC-X with CAC interacting with different amino acid molecules X.

| CAC-X systems | Bond lengths (Å) | |
|---|---|---|
| X= | $d_{H1\text{-}O1}$ | $d_{H2\text{-}O2}$ |
| A | 1.765 | 1.706 |
| D | 1.783 | 1.712 |
| E | 1.771 | 1.728 |
| G | 1.754 | 1.719 |
| I | 1.770 | 1.704 |
| K | 1.776 | 1.697 |
| L | 1.717 | 1.712 |
| M | 1.725 | 1.725 |
| N | 1.740 | 1.706 |
| Q | 1.804 | 1.700 |
| R | 1.804 | 1.728 |
| S | 1.773 | 1.702 |
| T | 1.771 | 1.677 |
| V | 1.761 | 1.708 |
| | $d_{\pi\text{-}\pi}$ | |
| F | 3.424 | |
| Y | 3.408 | |

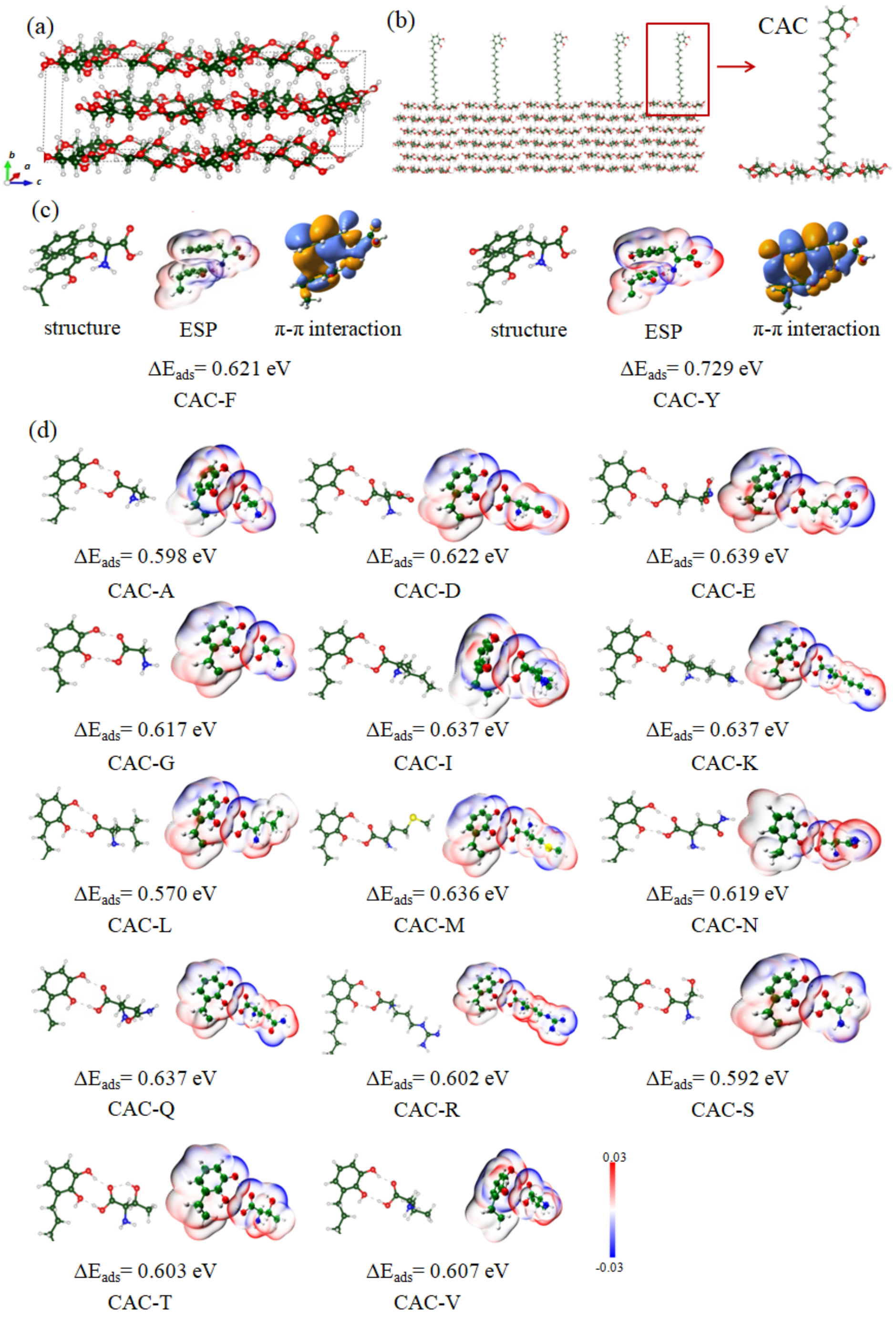


**Supplementary Figure 10.** DFT analysis. (a) Bulk structure of cellulose. (b) The structural model of modified cellulose structure with the alkyl chain containing fifteen carbon atoms in the skeleton and having the terminal linked with catechol. (c) Two optimized structures CAC-X (X= F and Y) with USO-g-gauze interacting with the amino acids X containing benzene ring and the corresponding adsorption energies ($\Delta E_{ads}$) as well as the electrostatic potentials and the correlative electron densities, where only the upper part of the structure is provided for clarity. (d) The remaining fourteen optimized structures CAC-X with CAC interacting with different amino acid molecules X, as well as the corresponding adsorption energies ($\Delta E_{ads}$) and the electrostatic potentials. The dark green, white, blue, red and yellow balls represent C, H, N, O and S atoms, respectively.

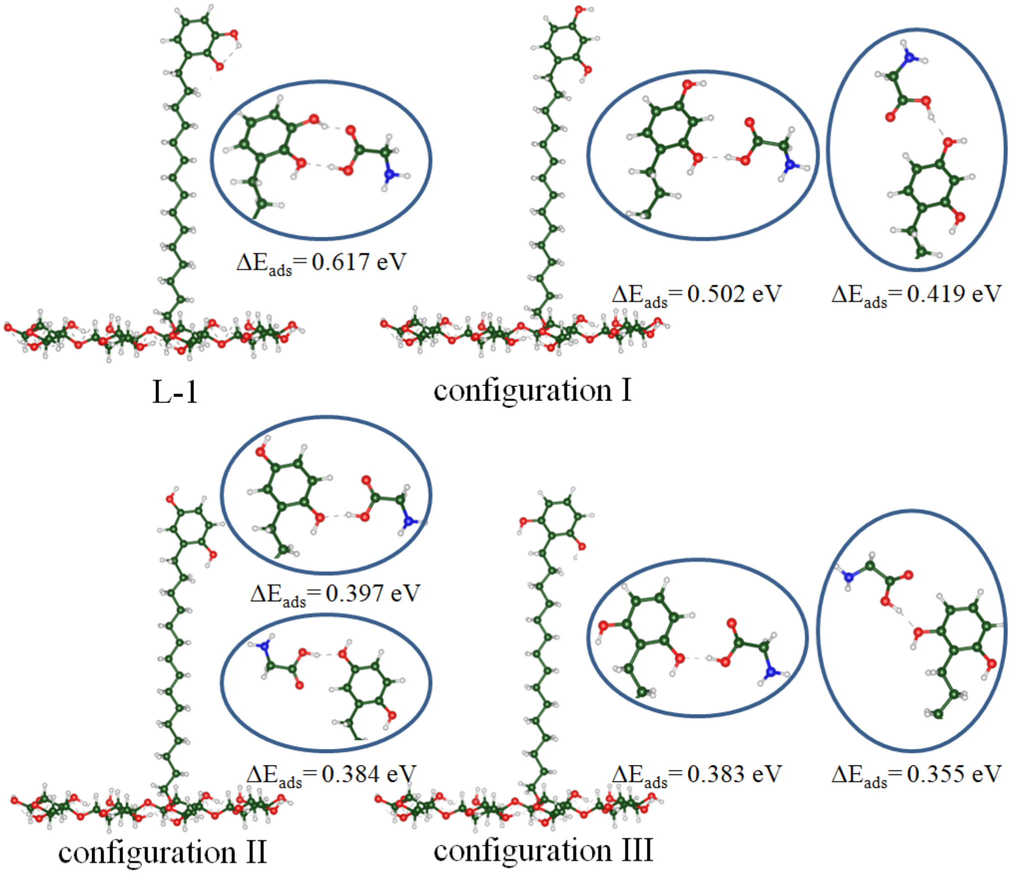


**Supplementary Figure 11.** The effect of relative position between two OH groups on the benzene ring on the adsorption energy by sampling amino acid molecule G interacting with the modified cellulose system.

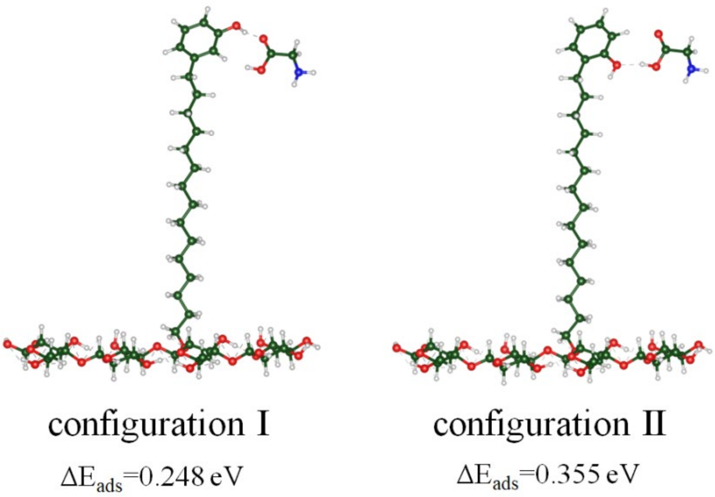


**Supplementary Figure 12.** The two correlative configurations for adsorbing the sampled amino acid molecule G on the modified cellulose system with the alkyl chain terminated by phenol with one OH group, and the corresponding adsorption energies.

11. Peeling force of gauzes on fresh wet rat femoral tissue

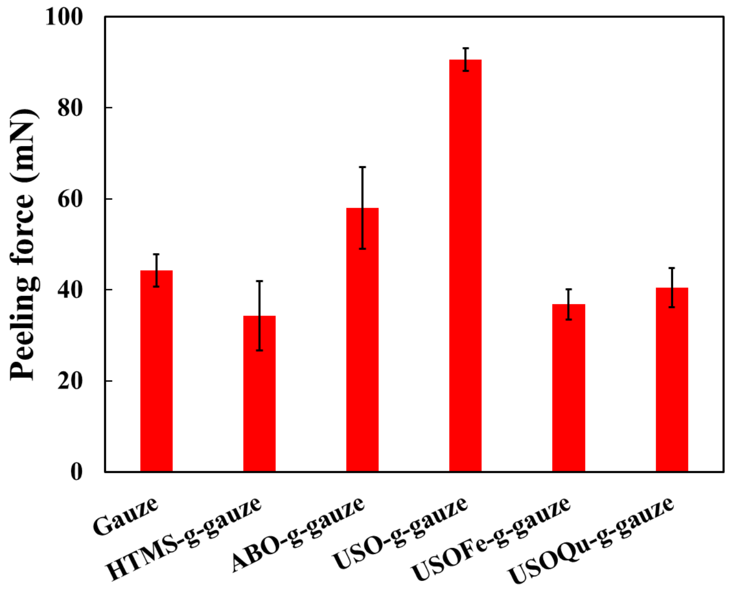


**Supplementary Figure 13.** Peeling force of gauzes on the rat femoral tissue. Data are shown as mean ± SD, *n* = 3, error bars represent SD. Source data are provided as a Source Data file.

12. Inactivation of catechol group on USO-g-gauze and their hemostatic performance

When USOFe-g-gauze is applied onto the rat femoral artery injury, blood spills (1s in Supplementary Fig. 15a, pointed by yellow arrow) and seeps out of the seam of gauze/wound surface (15s in Supplementary Fig. 15a, pointed by yellow arrow), it also diffuses throughout the whole four gauze layers in 15 s, but thereafter the blood-stained area on the top layer does not expand much, instead blood continuously oozes out from the gauze/tissue contact surface (Supplementary Fig. 15a). The gauze is removed 5 min later when the wound fully stops bleeding, re-bleeding is observed and much red fresh blood is around the wound (Supplementary Fig. 15a). Compared to USO-g-gauze, the hemostasis time and blood loss on this wound treated by USOFe-g-gauze significantly increases to ca. 289 s, and 1.32 g, respectively (Supplementary Figs. 15b, c). The instant static WCA of USOFe-g-gauze is 119° (higher than 68° of USO-g-gauze), but slowly reduces to 0° within 60 s with diffusion of water droplet

(Supplementary Fig. 14b), similar to that occurred for USO-g-gauze. Therefore, the weak adhesiveness of catechol-$Fe^{3+}$ groups to wet skin tissue, would be responsible for the substantially longer hemostatic time and more blood loss than USO-g-gauze and cotton gauze. Similar phenomena (blood diffusion, seepage, and re-bleeding) are found for the USOQu-g-gauze on the rat femoral artery injury (Supplementary Fig. 15a). This is also due to the less tissue adhesiveness of quinone groups existing on the USOQu-g-gauze surface. The hemostasis time and blood loss by USOQu-g-gauze are ca. 193 s and 0.87 g, respectively (Supplementary Figs. 15b, c).

The hemostatic performance of USOFe-g-gauze and USOQu-g-gauze on the rat liver laceration model is similar to that on the rat femoral artery injury model (Supplementary Fig. 16a). Blood flows down the liver and wets the gauze under the rat liver, which does not occur when USO-g-gauze is applied. On this injury, the hemostatic time and blood loss by USOFe-g-gauze are ca. 102 s and 0.48 g, respectively; and by USOQu-g-gauze are ca. 65 s and 0.28 g, respectively (Supplementary Figs. 16b, c). Thus, the catechol group is critical in controlling traumatic bleeding. The far superior hemostatic potential of USO-g-gauze to ABO-g-gauze (ABO has a three carbons alkyl chain) strongly suggests that a long hydrophobic alkyl chain is also of great importance. Therefore, USO, a catechol compound with a side alkyl chain having 15 carbons is a good candidate compound for surface modification of fabric gauze (such as cotton gauze and chitosan nonwoven) to prepare novel highly efficient hemostatic gauzes.

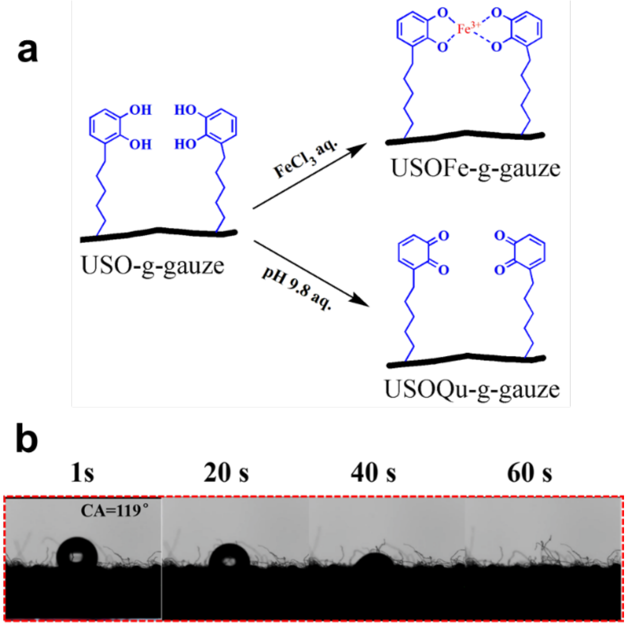


**Supplementary Figure 14.** Deactivation of catechol group on gauze. (a) Schematic diagram of the chelation reaction between catechol groups and $Fe^{3+}$ in the USOFe-g-gauze, and the transformation of catechol to quinone in pH 9.8 aq. solution for USOQu-g-gauze. (b) Water contact angle of USOFe-g-gauze with time.

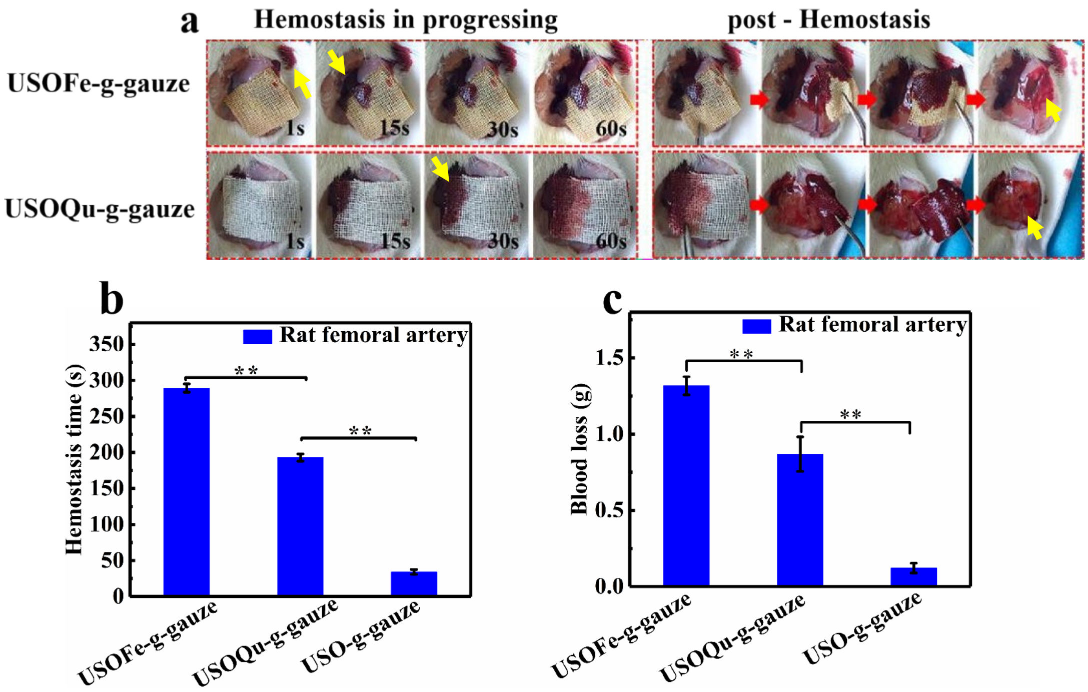


**Supplementary Figure 15.** Hemostatic efficacy of modified USO-g-gauze on rat femoral artery injury. (a) Gauze was put on the bleeding rat femoral artery injury, then it was removed from the wound after hemostatic state was reached. (b) Hemostatic time and (c) blood loss in the rat femoral artery injury model. Data in b and c are shown as mean ± SD, $n = 6$, error bars represent SD. Source data for b and c are provided as a Source Data file. $^{**}P < 0.01$, one-way analysis of variance (ANOVA).

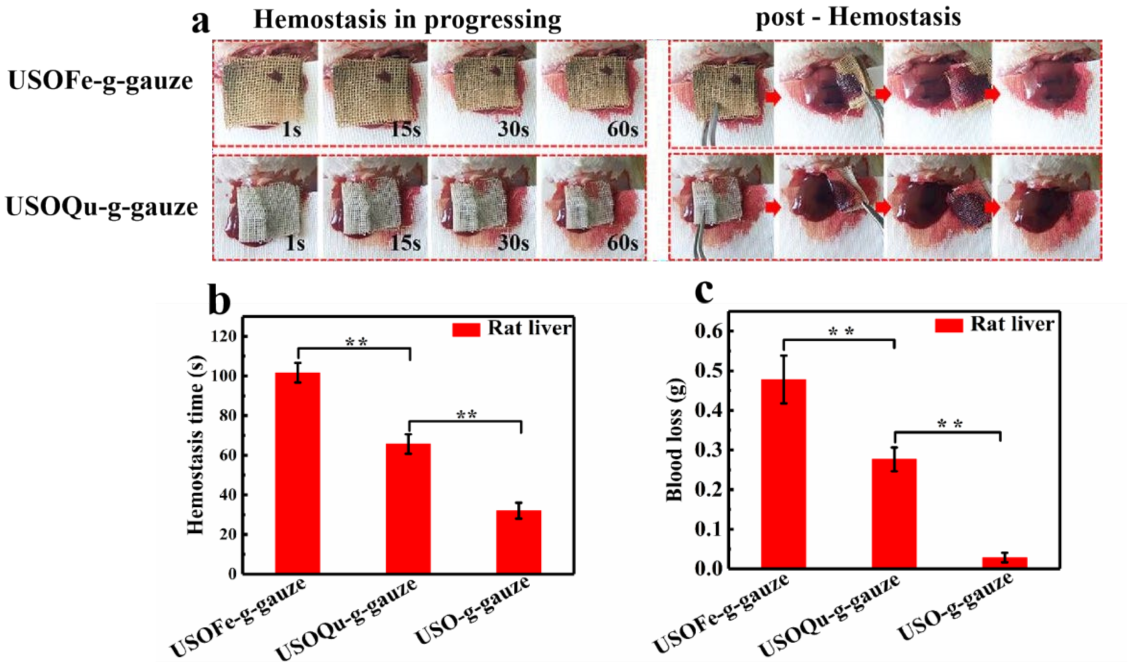


**Supplementary Figure 16.** Hemostatic efficacy of modified USO-g-gauze on rat liver. (a) Gauze was put on the bleeding rat liver injury, then it was removed from the wound after hemostatic state was reached. (b) Hemostatic time and (c) blood loss in the liver injury models. Data in b and c are shown as mean ± SD, $n = 6$, error bars represent SD. Source data for b and c are provided as a Source Data file. $^{**}P < 0.01$, one-way analysis of variance (ANOVA).

## 13. Morphology of Bandage®

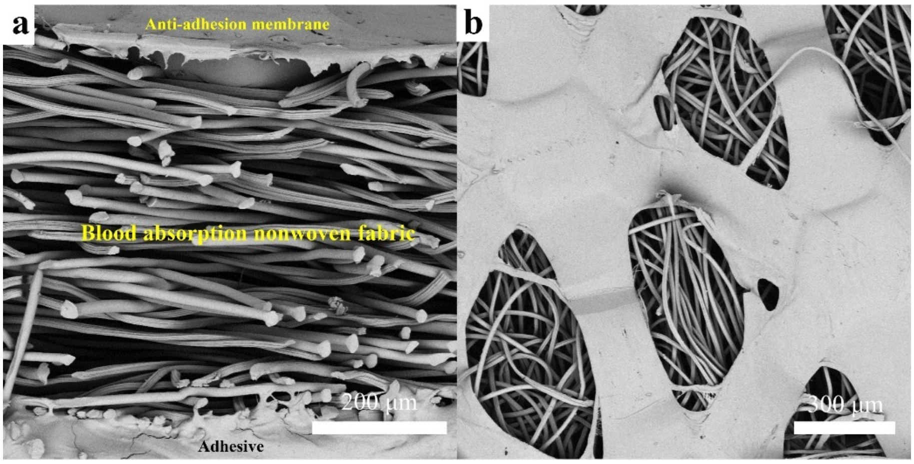


**Supplementary Figure 17.** SEM images of Bandage. (a) cross-section and (b) topology of a bandage. Three spots were observed independently with similar results.

## 14. Growth and proliferation of L929 on gauze samples

The growth and proliferation of L929 fibroblasts on USO-g-gauze are shown in Supplementary Fig. 18. On the gauze yarn, many green-dyed living fibroblasts with several red-dyed dead cells are observed. Obviously, the fibroblasts proliferate very

well with incubation time increasing from 1 to 3 d. The good cell biocompatibility of USO-g-gauze is comparable to that of cotton gauze and ABO-g-gauze (Supplementary Figs. 19a, b). The fibroblasts can also dwell and grow on HTMS-g-gauze, but the proliferation rate on this hydrophobic gauze is significantly smaller than that on the other three hydrophilic gauzes (Supplementary Fig. 19c). Therefore, USO-g-gauze can favor cell adhesion and proliferation, due to good balance of hydrophilicity/hydrophobicity and presence of catechol groups on this gauze.

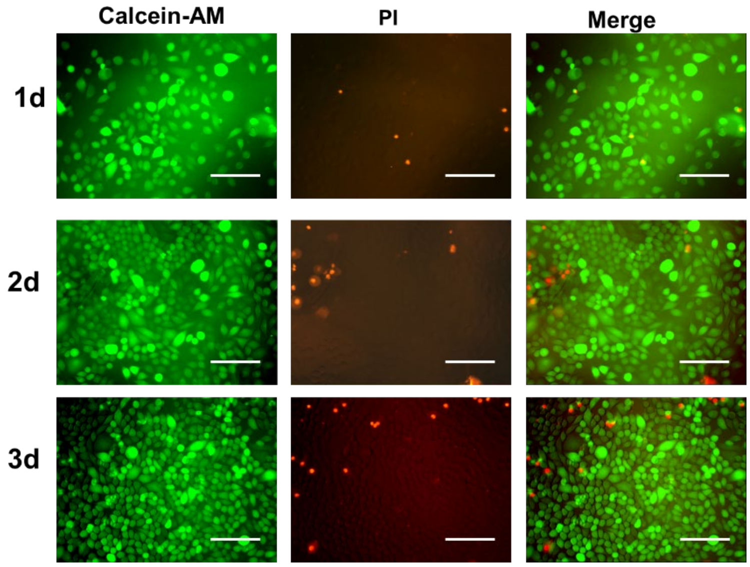


**Supplementary Figure 18.** Optical microscopy images of L929 fibroblast cells incubation with USO-g-gauze for 1, 2, and 3 d. Three spots were observed independently with similar results. Scale bar: 100 μm.

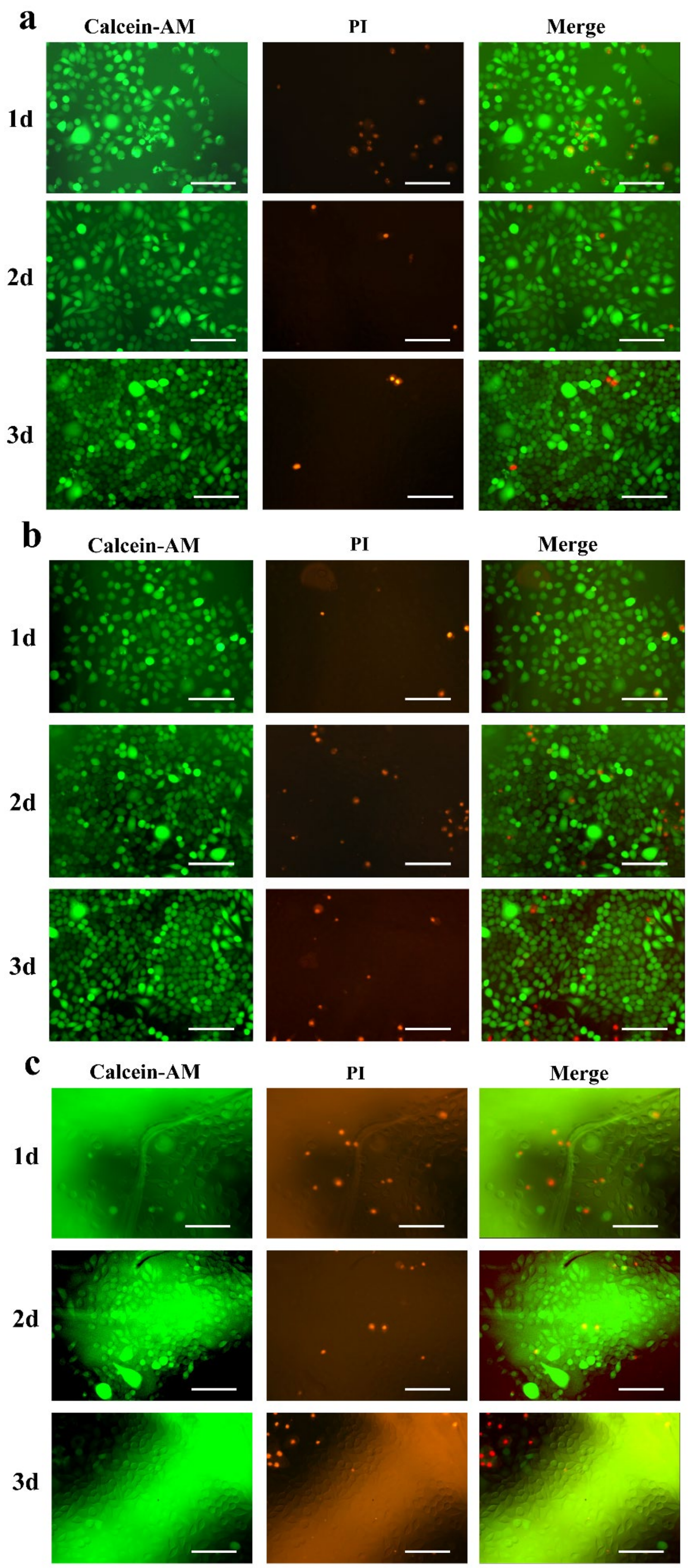


**Supplementary Figure 19.** Optical microscopy images of L929 cells incubation on (a) cotton gauze, (b) ABO-g-gauze, and (c) HTMS-g-gauze for 1, 2, and 3 d. Three spots were observed independently with similar results. Scale bar: 100 μm.

## Supplementary References


1. Ahn, B.K., Lee, D.W., Israelachvili, J.N. & Waite, J.H. Surface-initiated self-healing of polymers in aqueous media. *Nat. Mater.* **13**, 867-872 (2014).
2. Pertile, R.A.N., Andrade, F.K., Alves, C. & Gama, M. Surface modification of bacterial cellulose by nitrogen-containing plasma for improved interaction with cells. *Carbohydr. Polym.* **82**, 692-698 (2010).
3. Sun, X. et al. Chitosan/kaolin composite porous microspheres with high hemostatic efficacy. *Carbohydr. Polym.* **177**, 135-143 (2017).
4. Anitha, A. et al. Chitin and chitosan in selected biomedical applications. *Prog. Polym. Sci.* **39**, 1644-1667 (2014).
5. Perdew, J., Burke, K. & Ernzerhof, M. Generalized gradient approximation made simple. *Phys. Rev. Lett.* **77**, 3865-3868 (1996).
6. Grimme, S. Semiempirical gga-type density functional constructed with a long-range dispersion correction. *J. Comput. Chem.* **27**, 1787-1799 (2006).
7. Wu, X., Vargas, M., Nayak, S., Lotrich, V. & Scoles, G. Towards extending the applicability of density functional theory to weakly bound systems. *J. Chem. Phys.* **115**, 8748-8757 (2001).
8. Kresse, G. & Hafner, J. Ab initio molecular dynamics for liquid metals. *Phys. Rev. B* **47**, 558-561 (1993).
9. Kresse, G. & Hafner, J. Ab-Initio molecular-dynamics simulation of the liquid-metal amorphous-semiconductor transition in germanium. *Phys. Rev. B* **49**, 14251-14269 (1994).
10. Blochl, P. Projector augmented-wave method. *Phys. Rev. B* **50**, 17953-17979 (1994).
11. Li, W. & Neuhauser, D. Real-space orthogonal projector-augmented-wave method. *Phys. Rev. B* **102**, 195118 (2020).
12. Gessler, K. et al. β-D-Cellotetraose hemihydrate as a structural model for cellulose II. An X-ray diffraction study. *J. Am. Chem. Soc.* **117**, 11397-11406 (1995).
