# Supplementary material for: Efficient, biosafe and tissue adhesive hemostatic cotton gauze with controlled balance of hydrophilicity and hydrophobicity
Source: Nat Commun. 2022 Jan 27;13:552. doi: 10.1038/s41467-022-28209-8 (PMC8795195; doi:10.1038/s41467-022-28209-8)
Supplement: Supplementary file 3 — Description of Additional Supplementary Files [file 41467_2022_28209_MOESM3_ESM.docx]

**Description of Additional Supplementary Files**

**Supplementary Movie 1.** Water absorption dynamic of gauzes

**Supplementary Movie 2.** Non-compressing hemostasis by gauzes in rat femoral artery injury model.

**Supplementary Movie 3.** Compressing-hemostasis by HTMS-g-gauze and USO-g-gauze in rat femoral artery injury model.

**Supplementary Movie 4.** Compressing-hemostasis by chitosan and USO-g-chitosan gauzes in rat femoral artery injury model.

**Supplementary Movie 5.** Non-compressing hemostasis in rat liver injury model.

**Supplementary Movie 6.** Hemostasis by cotton gauze in pig femoral vein injury model.

**Supplementary Movie 7.** Hemostasis by USO-g-gauze in pig femoral vein injury model.
