# Supplementary material for: Efficient, biosafe and tissue adhesive hemostatic cotton gauze with controlled balance of hydrophilicity and hydrophobicity
Source: Nat Commun. 2022 Jan 27;13:552. doi: 10.1038/s41467-022-28209-8 (PMC8795195; doi:10.1038/s41467-022-28209-8)
Supplement: Supplementary file 11 — Reporting Summary [file 41467_2022_28209_MOESM11_ESM.pdf]

nature portfolio

Corresponding author(s): Haiqing Liu

Last updated by author(s): Nov 25, 2021

Reporting Summary

Nature Portfolio wishes to improve the reproducibility of the work that we publish. This form provides structure for consistency and transparency in reporting. For further information on Nature Portfolio policies, see our Editorial Policies and the Editorial Policy Checklist.

Statistics

For all statistical analyses, confirm that the following items are present in the figure legend, table legend, main text, or Methods section.

n/a	Confirmed	
☐	☒	The exact sample size (*n*) for each experimental group/condition, given as a discrete number and unit of measurement
☐	☒	A statement on whether measurements were taken from distinct samples or whether the same sample was measured repeatedly
☐	☒	The statistical test(s) used AND whether they are one- or two-sided<br>*Only common tests should be described solely by name; describe more complex techniques in the Methods section.*
☒	☐	A description of all covariates tested
☒	☐	A description of any assumptions or corrections, such as tests of normality and adjustment for multiple comparisons
☐	☒	A full description of the statistical parameters including central tendency (e.g. means) or other basic estimates (e.g. regression coefficient) AND variation (e.g. standard deviation) or associated estimates of uncertainty (e.g. confidence intervals)
☐	☒	For null hypothesis testing, the test statistic (e.g. *F*, *t*, *r*) with confidence intervals, effect sizes, degrees of freedom and *P* value noted<br>*Give P values as exact values whenever suitable.*
☒	☐	For Bayesian analysis, information on the choice of priors and Markov chain Monte Carlo settings
☒	☐	For hierarchical and complex designs, identification of the appropriate level for tests and full reporting of outcomes
☒	☐	Estimates of effect sizes (e.g. Cohen's *d*, Pearson's *r*), indicating how they were calculated

Our web collection on statistics for biologists contains articles on many of the points above.

Software and code

Policy information about availability of computer code

Data collection	No software was used.
Data analysis	Origin, Microsoft Excel

For manuscripts utilizing custom algorithms or software that are central to the research but not yet described in published literature, software must be made available to editors and reviewers. We strongly encourage code deposition in a community repository (e.g. GitHub). See the Nature Portfolio guidelines for submitting code & software for further information.

Data

Policy information about availability of data

All manuscripts must include a data availability statement. This statement should provide the following information, where applicable:

- Accession codes, unique identifiers, or web links for publicly available datasets
- A description of any restrictions on data availability
- For clinical datasets or third party data, please ensure that the statement adheres to our policy

All experimental data within the article and its Supplementary Information are available from the corresponding authors upon reasonable request. The source data of Figs. 2; 3c, d; 5b, c; 6b, c; 7e, f; 9c, d, Supplementary Figs. 3; 4a, b; 6o; 7c; 8a, b; 13; 15b, c; 16b, c and Supplementary Tables 1, 2, 3 are provided as a Source Data file.

Field-specific reporting

Please select the one below that is the best fit for your research. If you are not sure, read the appropriate sections before making your selection.

☒ Life sciences ☐ Behavioural & social sciences ☐ Ecological, evolutionary & environmental sciences

For a reference copy of the document with all sections, see nature.com/documents/nr-reporting-summary-flat.pdf

Life sciences study design

All studies must disclose on these points even when the disclosure is negative.

Sample size	1. n=6 (6 rats) for measuring blood loss and bleeding time on rat femoral artery injury model.<br>2. n=6 (6 rats) for measuring blood loss and bleeding time on rat liver laceration model.<br>3. n=3 (3 pigs) for measuring blood loss on pig femoral artery and skin injury model.<br>4. n=3 (3 rats) for peeling force of gauze on fresh rat femoral tissue.<br>5. n=6 (6 rats) for histological changes of the subcutaneous muscle tissue treated with gauzes.
Data exclusions	No data were excluded from the analysis.
Replication	1. Six rats were used for measuring blood loss and bleeding time on rat femoral artery injury model.<br>2. Six rats were used for measuring blood loss and bleeding time on rat liver laceration model.<br>3. Three pigs were used for measuring blood loss on pig femoral artery and skin injury model.<br>4. Three rats were used for peeling force of gauze on fresh rat femoral tissue.<br>5. 6 rats were used for histological changes of the subcutaneous muscle tissue treated with gauzes.<br>All these above replicates in each group were successful.
Randomization	Allocation of animals was random.
Blinding	Two investigators (He and Zhou) studied the blood loss and blood bleeding on animal injury models. They performed the hemostatsis evaluation in turn to minimize human error. The data were acquired by both of them. Data was analyzed firstly by Zhou, then verified by He, re-checked by Liu.

Reporting for specific materials, systems and methods

We require information from authors about some types of materials, experimental systems and methods used in many studies. Here, indicate whether each material, system or method listed is relevant to your study. If you are not sure if a list item applies to your research, read the appropriate section before selecting a response.

Materials & experimental systems

n/a	Involved in the study	
☒	☐	Antibodies
☐	☒	Eukaryotic cell lines
☒	☐	Palaeontology and archaeology
☐	☒	Animals and other organisms
☒	☐	Human research participants
☒	☐	Clinical data
☒	☐	Dual use research of concern

Methods

n/a	Involved in the study	
☒	☐	ChIP-seq
☒	☐	Flow cytometry
☒	☐	MRI-based neuroimaging

Eukaryotic cell lines

Policy information about cell lines

Cell line source(s)	L929 cell line was ordered from ATCC (Catalog No. C054)
Authentication	L929 cell line was used as received without authentication.
Mycoplasma contamination	L929 cell line was tested negative for mycoplasma contamination
Commonly misidentified lines (See ICLAC register)	No misidentified cell line was used

Animals and other organisms

Policy information about studies involving animals; ARRIVE guidelines recommended for reporting animal research

Laboratory animals	Sprague-Dawley rat(6-7 weeks old; Male); Bama miniature pig (2 months old ; Male).
Wild animals	Not involved wild animals
Field-collected samples	Not involve sample collected from fields
Ethics oversight	Animal Ethics Committee of Fujian Normal University （Protocol No. IACUC-20180013）

Note that full information on the approval of the study protocol must also be provided in the manuscript.
